# Ultrasonographic study of the orbit as an early diagnostic tool in Vogt Koyanagi Harada disease (VKH): A case report

**DOI:** 10.1016/j.heliyon.2024.e26196

**Published:** 2024-02-10

**Authors:** José Fernández-Navarro, Jorge García-García, José Gregorio García-García, Tomás Segura

**Affiliations:** a Department of Neurology, Puerta del Mar Universitary Hospital, av/Ana de Viya 21, 11009 Cádiz, Spain; b Department of Neurology, Complejo Hospitalario Universitario de Albacete, c/Hermanos Falco 37, 02006 Albacete, Spain; c Department of Ophthalmology, Complejo Hospitalario Universitario de Albacete, c/Hermanos Falco 37, 02006 Albacete, Spain; d Instituto de Investigación en Discapacidades Neurológicas (IDINE), Facultad de Medicina, Universidad de Castilla-La Mancha, 02008 Albacete, Spain

**Keywords:** Thickening of the choroid, Vogt koyanagi Harada, Immunosuppressive therapy, Early diagnostic, Echographic features

## Abstract

In recent years, ultrasound has demonstrated its usefulness in the approach to vascular structures and other tissues such as the orbit, facilitating the early diagnosis of various diseases without having to rely on other more invasive or less available tests. In Vogt Koyanagi Harada syndrome, characterised by bilateral acute uveitis, ocular ultrasound is a clear example of the usefulness of ultrasonography in early diagnosis, facilitating the initiation of specific treatment to change the ominous natural history of this disease. This case shows the usefulness of the echography to make the differential diagnosis with other diseases that clinical onset could be similar than VKH, but with a different diagnostic and therapeutic approach.

## 1 Introduction

Vogt Koyanagi Harada syndrome (VKH) is a granulomatous inflammatory disease of autoimmune origin with predominantly ocular involvement in the form of bilateral acute uveitis. Its pathophysiology consists of a T-lymphocyte-mediated autoimmune response against melanocytes present mainly in the uveal, cutaneous, meningeal and auditory tissues [1,2]. It therefore has a special predilection for pigmented races, particularly Asian and Hispanic Americans, and is less common in Caucasians [[1], [2], [3], [4]].

In VKH, ocular symptoms are predominant, being characteristic the development of a severe bilateral visual deficit, with papillary oedema and the presence of an exudative retinal detachment in the ocular fundus which it is high representative of this syndrome. However, this finding is usually late in onset and could cause a delay in diagnosis and treatment leading to a worse outcome and irreversible visual loss. This delay more frequent when the initial symptoms are non-specific, such as headache or tinnitus, and the fundoscopic examination only shows papillary oedema, which can be interpreted as intracranial hypertension of unclear aetiology [5,6].

Imaging tests can be used to facilitate the diagnosis of VKH, such as fluorescein angiography which often shows characteristic findings that allow the diagnosis. However, in the presence of opaque media (cataracts, synechiae or dense vitritis), characteristics features may be either absent or difficult to visualize them. Advances in optical coherence tomography (OCT) have allowed a quick and noninvasive means of detailed chorioretinal morphologic evaluation. OCT findings in early-stage VKH include choroidal thickening, serous retinal detachments and septate fluid loculations with inflammatory products in subretinal space. However, this technique is less accessible and it is not routinely performed in the emergency department unlike the ultrasonography study.

Thus, we would like to highlight the usefulness of orbital ultrasound to make an early diagnosis in cases of VKH.

## 2 Case description

A 42-year-old woman, with no history of trauma or ocular surgery, whose symptoms began with insidious onset of oppressive bifrontal headache of moderate intensity, which partially subsided with analgesia but interrupted her sleep at night. The headache was associated with nausea as well as vomiting and photophobia at the moments of greatest intensity. She also reported decreased bilateral visual acuity and the development of persistent tinnitus.

**Fig. 1:**
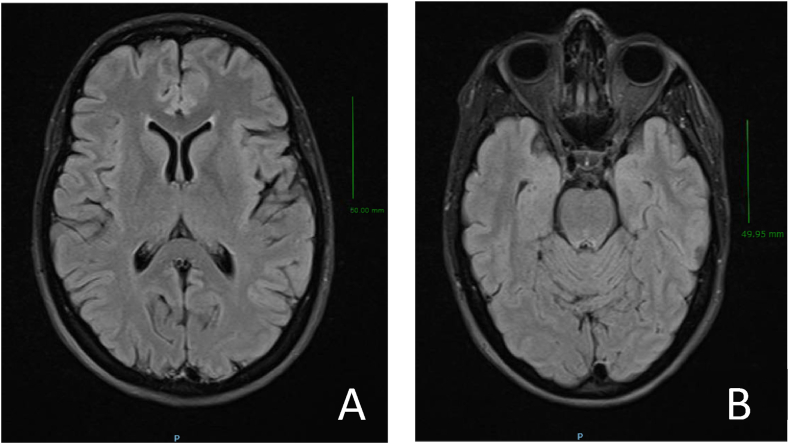
Magnetic resonance imaging (MRI, scale bar 5 cm) of the brain (A) and orbits (B).

The initial examination showed no focal neurological deficit, and the fundus showed a significant bilateral papillary elevation, suggestive of papillary oedema. Visual acuity of the right eye (RE) was 0.7 and of the left eye (LE) 0.6. Additional tests (brain MRI with contrast and laboratory tests) revealed no relevant findings (Fig. 1A and 1.B).

**Fig. 2:**
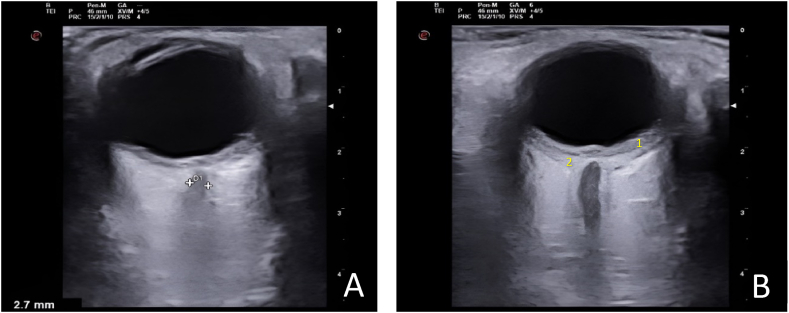
***Orbital ultrasound at the time of admission*.** It shows a diffuse posterior choroidal thickening [left eye (B) > right eye (A)] together with an accumulation of subretinal fluid. These ultrasound findings suggested the existence of bilateral chorioretinitis: o *1.Diffuse posterior choroidal thickening* o *2.An accumulation of subretinal fluid*.

A syndromic diagnosis of intracranial hypertension (ICHT) was established. It was decided to complete the study with orbital ultrasound (Fig. 2A and 2.B), which revealed diffuse posterior choroidal thickening (left > right) together with an accumulation of subretinal fluid without the presence of thickening of the optic nerve sheath or papillary protrusion that would suggest true ICHT. These ultrasound findings suggested the existence of bilateral chorioretinitis and it was decided to admit the patient for further investigation. Because of her symptom (headaches, nausea, general malaise …), a lumbar puncture (LP) was performed to complete the aetiological study, showing lymphocytic pleocytosis and hyperproteinorrachia, with normal opening pressure and multiple PCRs and Gram stains proving negative.

Serological tests for Treponema pallidum as well as immunological, microbiological for bacteria/mycobacteria and anatomopathological studies were negative. Clinical debut, negative analytics and pleocytosis in cerebrospinal fluid suggested a aseptic meningitis.

**Fig. 3:**
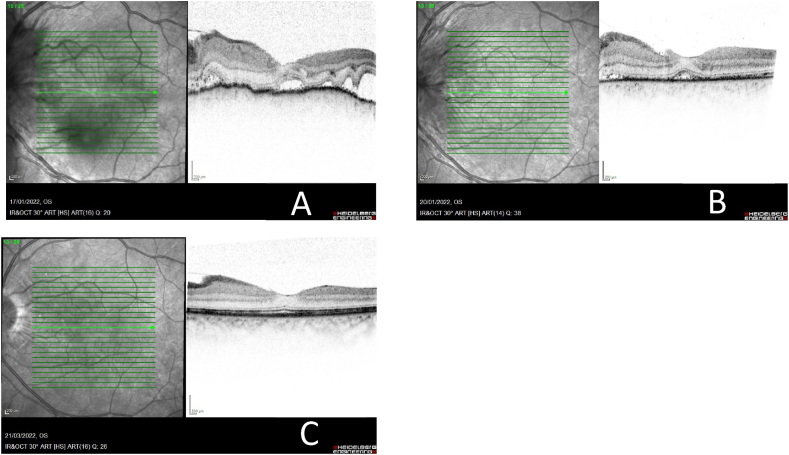
Early-stage Vogt Koyanagi Harada disease. **OCT diagnostic image of left eye at the time of admission pre – treatment (A), post – treatment after 7 days (B) and on subsequent control at 2 months (C).** A) Multiple serous detachments of the neuroepithelium with accumulation of subretinal fluid and choroidal thickening. B) and C) Resolution of the retinal detachments without alterations can be clearly seen in the OCT of the left eye at two months after the initiation of treatment with corticoids. The choroid present a normal pattern and thickness.

Optical coherence tomography (OCT) showed a very manifest overelevation in both eyes (LE > RE), with no clear edematous aspect, rather of an infiltrative/inflammatory type, with subretinal fluid infiltrate (Fig. 3A). Definitely, OCT confirmed the ocular ultrasound findings, showing significant choroidal thickening in both eyes.

Given the clinical characteristics of the patient together with the ultrasound findings suggestive of chorioretinitis confirmed by the ophthalmological study, as well as the presence of aseptic meningitis, once other inflammatory/infiltrative processes had been ruled out, a diagnosis of VKH syndrome was made. Appropriate treatment was started early with boluses of methylprednisolone 1 g/day for five days associated with immunosuppressive treatment with weekly methotrexate and adalimumab 40 mg every other week.

**Fig. 4:**
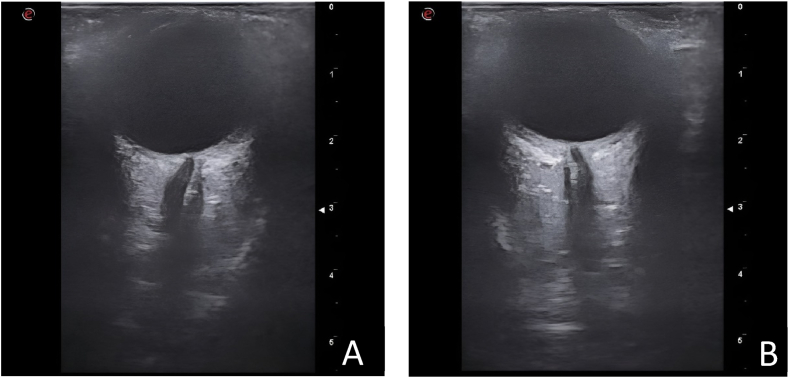
***Orbital ultrasound at* 12 months*.*** Ultrasonographic study of the orbit confirmed resolution of the choroidal thickening in the right eye (A) and almost complete resolution in the left eye (B) without the presence papillary protrusion.

On subsequent examination, the patient showed excellent clinical improvement, with complete visual recovery and orbital ultrasound (Fig. 4A and 4.B) confirmed resolution of the choroidal thickening in the right eye and almost complete resolution in the left eye. A control examination of the fundus showed no evidence of papillary oedema. Post-treatment control OCT (fig. 3B and 3.C) confirmed improvement without subsequent subretinal fibrosis and an absence of evident synechiae. Visual acuity of the right eye was 1.0 and of the left eye was 0.9 after high doses of corticosteroids.

## 3 Discussion

In cases of VKH, orbital ultrasound may show very suggestive findings such as diffuse thickening (often more striking in the peripapillary region) of the posterior choroid bilaterally together with a multifocal exudative retinal detachment [1,6,7]. These findings facilitate an early diagnosis of crucial importance because a diagnostic and therapeutic delay in VKH determines progression to a chronic recurrent stage leading to a worse prognosis with a risk of permanent visual loss. For this reason, during the prodromal phase and, on some occasions, the uveitic phase, in which the urgent ophthalmological study concentrating on the fundus can provide little diagnostic information, the possibility of performing an orbital ultrasound scan could help us to successfully tackle this clinical entity. This allows an early therapeutic approach and prevents choroidal inflammation from leading to irreversible ocular complications [8,9].

The natural history of VKH syndrome is divided into four stages: prodromal, acute uveitic, convalescent and chronic/recurrent. As in our case, prodromal phase simulate a systemic viral picture with neurological manifestations. The acute uveitic phase can last several weeks and is characterised by diffuse choroiditis and papillary oedema as the earliest findings. Without adequate treatment this phase gives rise to a chronic recurrent phase with recurrence of granulomatous uveitis and other complications such as retinal fibrosis. From all the clinical changes that occurred in the late stages of VKH, subretinal fibrosis is the only one perceptible for ocular echography. The impact of subretinal fibrosis on the disease refers to the poor and irreversible visual acuity [5].

Ocular ultrasound has also showed its usefullness in VKH disease from distinguishing differential diagnosis like intracranial hypertension, presenting a very similar clinical onset with headache and visual deficit. Thus, we would like to highlight the usefulness of orbital ultrasound in cases of VKH because in setting of emergency departments, ecography is an available tool with an acceptable sensitivity and specificity. The main limitation of the ocular ultrasound is that reliability and quality of scans depend on physician experience. The early inflammatory changes cause consistent echographic findings included a diffuse thickening of the choroid posterior or serous retinal detachment while late manifestations include ocular depigmentation and chorioretinal depigmented scars.

Latter criteria are based on compliance with specific clinical data supported by complementary tests such as orbital ultrasound and optical coherence tomography (OCT), once other causes of uveomeningitis (sympathetic ophthalmia - ocular trauma/surgery -, Behçet's disease, choroidal lymphoma) have been excluded. Other types of uveitis can also mimic the VKH syndrome like sarcoidosis, syphilis and tuberculosis, may result in infiltration of the choroid. However, these entities tend to show a more nodular or multifocal pattern of choroiditis than does VKH syndrome.

The initial diagnostic criteria for VKH [10] were limited, mostly resulting in very late diagnoses. For this reason, in 2021 the Standardisation of Uveitis Nomenclature (SUN) Working Group, following the same diagnostic criteria proposed in 2018 by Yang P et al. [11,12], abandoned the previous terminology and developed more specific criteria that would allow an adequate early diagnosis [13]. Criteria for early-stage VKH disease are showed in Supplementary Table 1.

Treatment of VKH consists of early administration of corticosteroids to limit disease progression, but there is no clear consensus on the optimal route of administration and dose. Recent publications have shown that high doses of corticosteroids, compared to lower doses, decrease disease activity, reduce recurrences and limit late-stage progression [14].

Finally, along with corticosteroid therapy, recent observational articles support starting from the beginning with immunosuppressive biologic agents, such as adalimumab, as they have been shown to provide a better visual prognosis by significantly decreasing progression to the chronic recurrent phase, in particular by permitting the withdrawal or reduction corticosteroid doses [15]. Thus, the visual prognosis is usually excellent in those patients with an early diagnosis together with an aggressive and sustained therapeutic approach, thus reducing late visual morbidity.

## 4 Conclusions

VKH is a bilateral granulomatous panuveitis that can have a great impact on the patient's life with clear personal and occupational repercussions. Visual involvement and ocular complications will largely depend on early diagnosis as well as its therapeutic aggressiveness.

The recent update of diagnostic criteria allows patients to be treated at an early stage, leading to a better prognosis. In the frequent cases of prodromal presentation or in cases with greater diagnostic difficulty, orbital ultrasound can show characteristic ultrasonographic findings, guiding the diagnosis and allowing early treatment to be initiated. In addition, it appears that ultrasound may play a role in monitoring subclinical choroiditis and quantifying the degree of inflammatory activity, determining escalation to more potent immunotherapy.

## Funding

The APC was funded by the Instituto de Investigación en Discapacidades Neurológicas (IDINE).

## Informed consent statement

Not applicable.

## Ethical considerations

The authors declare that no human or animal experiments were conducted as part of this study.

## Data availability statement

Data included in article.

## CRediT authorship contribution statement

**José Fernández-Navarro:** Writing – review & editing, Writing – original draft, Visualization, Validation, Supervision, Software, Resources, Project administration, Methodology, Investigation, Funding acquisition, Formal analysis, Data curation, Conceptualization. **Jorge García-García:** Writing – review & editing, Writing – original draft, Visualization, Validation, Supervision, Software, Resources, Project administration, Methodology, Investigation, Funding acquisition, Formal analysis, Data curation, Conceptualization. **José Gregorio García-García:** Writing – review & editing, Writing – original draft, Visualization, Validation, Software, Resources, Project administration, Methodology, Investigation, Funding acquisition, Formal analysis, Data curation, Conceptualization. **Tomás Segura:** Writing – review & editing, Writing – original draft, Visualization, Validation, Supervision, Software, Resources, Project administration, Methodology, Investigation, Funding acquisition, Formal analysis, Data curation, Conceptualization.

## Declaration of competing interest

The authors declare that they have no known competing financial interests or personal relationships that could have appeared to influence the work reported in this paper.
