# Supplementary material for: Ultrasonographic study of the orbit as an early diagnostic tool in Vogt Koyanagi Harada disease (VKH): A case report
Source: Heliyon. 2024 Feb 10;10(4):e26196. doi: 10.1016/j.heliyon.2024.e26196 (PMC10877363; doi:10.1016/j.heliyon.2024.e26196)
Supplement: Multimedia component 1 [file mmc1.pdf]

Table 1. Criteria for early-stage VKH disease

**1. Evidence of Harada disease**<br>A. Exudative retinal detachment AND (B. and/or C.)<br>B. Multiloculated appearance on fluorescein angiogram<br><br>OR<br><br>C. Septae on optical coherence tomogram<br><br>**OR**
**2. Panuveitis with at least two of the following neurologic symptoms or signs:**<br>• Headache<br>• Tinnitus<br>• Dysacusis<br>• Meningismus<br>• Cerebrospinal fluid pleocytosis
**AND**
**3. No history of penetrating ocular trama or vitreoretinal surgery prior to disease onset**
Exclusions<br>○ *Positive serology for syphilis using a treponemal test*<br>○ *Evidence for sarcoidosis (either bilateral hilar adenopathy on chest imaging or tissue biopsy demonstrating noncaseating granulomata)*
